# Fifteen Novel *EIF2B1-5* Mutations Identified in Chinese Children with Leukoencephalopathy with Vanishing White Matter and a Long Term Follow-Up

**DOI:** 10.1371/journal.pone.0118001

**Published:** 2015-03-11

**Authors:** Haihua Zhang, Lifang Dai, Na Chen, Lili Zang, Xuerong Leng, Li Du, Jingmin Wang, Yuwu Jiang, Feng Zhang, Xiru Wu, Ye Wu

**Affiliations:** 1 Department of Pediatrics, Peking University First Hospital, Beijing, China; 2 School of Life Sciences, Fudan University, Shanghai, China; Huashan Hospital, Fudan University, CHINA

## Abstract

Leukoencephalopathy with vanishing white matter (VWM) is one of the most prevalent inherited childhood white matter disorders, which caused by mutations in each of the five subunits of eukaryotic translation initiation factor 2B (*EIF2B1-5*). In our study, 34 out of the 36 clinically diagnosed children (94%) were identified to have *EIF2B1-5* mutations by sequencing. 15 novel mutations were identified. CNVs were not detected in patients with only one mutant allele and mutation-negative determined by gene sequencing. There is a significantly higher incidence of patients with *EIF2B3* mutations compared with Caucasian patients (32% vs. 4%). c.1037T>C (p.Ile346Thr) in *EIF2B3* was confirmed to be a founder mutation in Chinese, which probably one of the causes of the genotypic differences between ethnicities. Our average 4.4 years-follow-up on infantile, early childhood and juvenile VWM children suggested a rapid deterioration in motor function. Episodic aggravation was presented in 90% of infantile cases and 71.4% of childhood cases. 10 patients died during the follow-up. The Kaplan-Meier curve showed that the median survival time is 8.83 ± 1.51 years. This is the largest sample of children in a VWM follow-up study, which is helpful for a more depth understanding about the natural course.

## Introduction

Leukoencephalopathy with vanishing white matter (VWM, OMIM306896) is one of the most prevalent inherited leukoencephalopathies in children [1,2]. Inherited in an autosomal recessive manner, VWM is the first known hereditary human disease caused by direct defects in the initiation of the protein translation process, specifically, gene defects in *EIF2B1-5*, encoding the five subunits of eukaryotic translation initiation factor 2B (eIF2Bα, β, γ, δ and ε) [3,4]. VWM is a progressive disorder clinically dominated by progressive cerebellar ataxia and spasticity. Cognition is relatively preserved, and optic atrophy with loss of vision and epilepsy might occur [5–7]. VWM has an extremely wide phenotypic variation and might affect people of all ages, including antenatal, infantile, early childhood, juvenile and adult [8–10]. The most common type of VWM is early childhood type. To date, more than 250 cases and 150 mutations of *EIF2B1-5* have been reported [11][12].

Since we reported the first Chinese case of VWM in 2006, a total of 34 Chinese cases have been identified with *EIF2B1-5* mutations. Our previous studies have revealed a unique *EIF2B1-5* mutation spectrum in Chinese patients, with much higher *EIF2B3* incidence than Caucasian population [13]. In this study, we further analyzed the genotype of the 34 Chinese children with VWM, and 15 novel mutations were identified. Natural history was followed up for an average 4.4 years.

## Materials and Methods

### Patients

**Fig 1:**
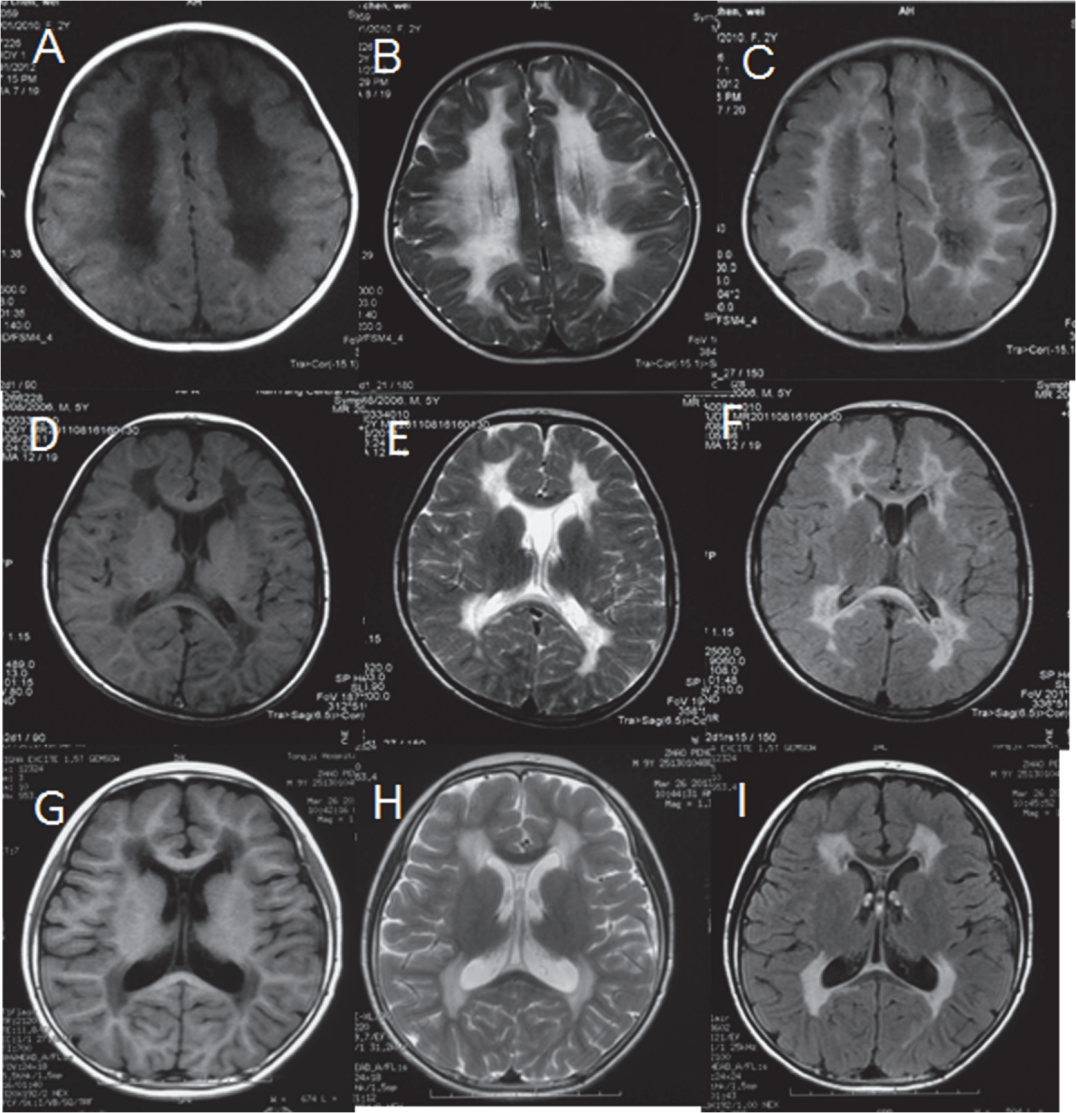
Brain MRI in VWM children. MRI from an infantile case (**A, B, C**), an early childhood case (**D, E, F**) and a juvenile case (**G, H, I**) respectively. In infantile and early childhood case, diffuse and symmetrical low signal in T1 (A, D), high signal in T2 (B, E) and Flair (C, F) images were shown in cerebral white matter, with part of white matter becomes CSF-like signal (low signal in Flair images). The white matter involvement in the juvenile case is milder, with only periventricular white matter and inner rim of corpus callosum involved and without CSF-like changes.

The clinical diagnosis of early childhood VWM (onset age 2–6years) is mainly on the basis of the following criteria [14]. 1) Early psychomotor development is usually normal or mildly delayed; 2) The clinical presentation consists of early-childhood onset of episodic and chronically progressive neurological regression. Episodes of rapid deterioration might be provoked by mild head trauma, febrile infection or fear; 3) Neurological signs mainly consist of cerebellar ataxia and spasticity; and 4) The brain MRI shows diffuse and symmetrical signal in cerebral white matter similar to cerebrospinal fluid on T1-weighted, T2-weighted and flair images (Fig. 1). The diagnosis of infantile (onset before 2 year-of-age) and juvenile (onset later than 6 year-of-age) patients was based on progressive motor regression and brain MRI [15]. Thirty-six clinically diagnosed cases were collected from September 2006 to December 2013 in Peking University First Hospital. All cases were Han Chinese.

This study was approved by the clinical research ethics committee of Peking University First Hospital. Written informed consent was obtained from all of the families on behalf of the children enrolled.

### Genotype analysis

#### EIF2B1-5 gene sequencing

Genomic DNA was extracted from the white blood cells of the children and their parents. The sequence of *EIF2B1-5* was obtained from the UCSC Genome Bioinformatics database (*EIF2B1*: NM_001414; *EIF2B2*:NM_014239; *EIF2B3* NM_020365; *EIF2B4*:NM_172195; *EIF2B5*: NM_003907). The exons and flanking introns were amplified. The novel mutations identified in patients were tested in 100 controls. The pathogenicity of the novel missense mutations were predicted by Software of PolyPhen2, SIFT and Mutation Taster.

#### CNV analysis

To further analyze the patients with negative sequencing results (including the patients with one mutant allele), Affymetrix SNP 5.0 chip technology was used to detect the presence of copy number variation (CNV), especially the deletions covering the *EIF2B1-5*.

#### Founder mutation determination

**Fig 2:**
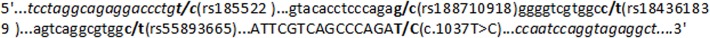
Primers in allele-specific PCR. The forward primer (in italics) ended either in T or C (rs185522), and a common backward primer (in italics) was used. The length of the PCR products was 1.8 Kb. Allele-specific PCR was performed to amplify fragments containing rs188710918, rs184361839 rs55893665, and c.1037.

Among the 11 patients carrying mutations in *EIF2B3*, 8 (73%) patients harbored at least one copy of the p.Ile346Thr (c.1037T>C) mutation. The nature of the founder mutation was suspected and determined by allele-specific PCR. Fifty-nine SNPs within 2.2 Kb upstream and downstream of the c.1037 were genotyped, and 4 informative SNPs (rs185522, rs188710918, rs184361839, and rs55893665) were found. The forward primer ended either in T or C (rs185522), and a common backward primer was used (Fig. 2). The forward primers used were 5'tcctaggcagaggaccctgt3' or 5'tcctaggcagaggaccctgc3', and the reverse primer was 5'tgagcctctacctgga ttgg 3'. The length of the PCR products was 1.8 Kb. Allele-specific PCR was performed to amplify fragments containing rs188710918, rs184361839 rs55893665, and c.1037. Single alleles containing either the wild type or mutant c.1037 were amplified. To determine whether c.1037T>C is a founder mutation, SNPs within the amplified mutant alleles from the patients were compared.

### Follow-up study

#### Methods of the follow-up

Thirty-four cases confirmed by gene sequencing were followed-up. Since 2006 when the first VWM child was diagnosed, the number of patients gradually increased. The follow-up was conducted at 2008, 2010 and 2013 respectively. The first time follow-up, second time follow-up and third time follow-up rates were 91.67% (11/12), 82.35% (14/17), and 94.12% (32/34), respectively. During the follow-up, patients were interviewed by telephone (80%), home visits, or outpatient visiting. The analysis focused mainly on the progression of motor impairment, seizures, episodic aggravation, and survival rate. Given the limitations of the follow-up methods, we could not objectively carry on cognitive evaluation for children. Therefore we failed to conduct a detailed assessment of cognitive impairment. For the children who died during the follow-up period, each visit after the death was counted as being followed.

The assessment of motor function was based on the Gross Motor Function Classification System (GMFCS) for cerebral palsy, Chinese version [16]. Levels I-V were classified according to motor function and age. For the patients who acquired the ability to walk, level I was classified as walking independently indoors and outdoors, level II was classified as walking independently but with limitations on uneven surfaces and inclines, level III was classified as walking with assistance, level IV was classified as ambulance using a power wheelchair, and level V was classified as the loss of independent mobility.

#### Statistics

Each patient had an initial visit and 1–3 follow-up visits. In the analysis and description of motor progression with prolonged disease duration, according to the disease duration of the four time points mentioned above, we divided the children into four groups: disease duration < 3 years, 3–5 years, 5–8 years, and more than 8 years. If the follow-up data at different times for a child crossed groups, the final follow-up results were used.

For survival analysis, age of disease onset in the children was used as the starting point. For children who died during the follow-up period, the death time was the end point. The last follow-up was the end point for the patients being alive. We used a Kaplan-Meier curve to describe the life span, and the statistical analyses of the data were conducted using SPSS16.0 software.

## Results

### Genotype features of Chinese VWM children

#### Overall findings

34 out of the 36 children clinically diagnosed (34/36, 94%) were identified to have *EIF2B1-5* mutations by gene sequencing. The patients were compound heterozygous or homozygous, with the mutations inherited from their parents with normal phenotypes. In two patients, mutations were identified in only one allele. Two patients were mutation-negative. Thirty-seven mutations were found in *EIF2B1-5*, including 33 missense mutations, 1 nonsense mutation, and 3 deletions (1 in-frame and 2 frame-shift mutations) (Table 1). 15 novel mutations were identified. Novel mutations were not found in 100 controls. CNVs were not detected by aCGH in patients with only one mutant allele and mutation-negative determined by gene sequencing.

**Table 1:** *EIF2B1-5* mutations in 34 gene-confirmed VWM patients.

Case	Phenotype	Gene	Exons	Nucleotide change	Amino-acid change	Novel / reported	Parental derivation	Pathogenicity Prediction		
								PolyPhen2 (Score)	SIFT (Score)	MutationTaster
1	early childhood	*EIF2B3*	2	c.140G>A	p.Gly47Glu	reported	paternal	/	/	/
			9	c.1037T>C	p.IIe346Thr	reported	maternal	/	/	/
2	infantile	*EIF2B5*	7	c.943C>T	p.Arg315Cys	reported	maternal	/	/	/
			7	c.943C>T	p.Arg315Cys	reported	paternal	/	/	/
3	infantile	*EIF2B5*	7	c.1126A>G	p.Asn376Asp	reported	maternal	/	/	/
			9	c.1340C>T	p.Ser447Leu	reported	paternal	/	/	/
4	early childhood	*EIF2B5*	6	c.805C>T	p.Arg269*	reported	maternal	/	/	/
			7	c.1004G>C	p.Cys335Ser	reported	/	/	/	/
5	early childhood	*EIF2B3*	7	c.674G>A	p.Arg225Gln	reported	maternal	/	/	/
			7	c.674G>A	p.Arg225Gln	reported	paternal	/	/	/
6	juvenile	*EIF2B5*	1	c.185A>C	p.Asp62Val	reported	paternal	/	/	/
			7	c.1016G>C	p.Arg339Pro	reported	maternal	/	/	/
7	early childhood	*EIF2B5*	8	c.1157G>T	p.Gly386Val	reported	maternal	/	/	/
			13	c.1827–1838del	p.S610-D613del	reported	/	/	/	/
8	early childhood	*EIF2B3*	9	c.1037T>C	p.IIe346Thr	reported	paternal	/	/	/
			9	c.1037T>C	p.IIe346Thr	reported	/	/	/	/
9	infantile	*EIF2B3*	9	c.1037T>C	p.IIe346Thr	reported	maternal	/	/	/
			/	/	/	/	/	/	/	/
10	early childhood	*EIF2B5*	3	c.337C>A	p.Arg113Cys	reported	maternal	/	/	/
			6	c.806G>A	p.Arg269Gln	reported	paternal	/	/	/
11	early childhood	*EIF2B3*	9	c.1037T>C	p.IIe346Thr	reported	maternal	/	/	/
			9	c.1037T>C	p.IIe346Thr	reported	paternal	/	/	/
12	infantile	*EIF2B2*	2	c.254T>A	p.Val85Glu	reported	paternal	/	/	/
			8	c.922G>A	p.Val308Met	reported	maternal	/	/	/
13	infantile	*EIF2B4*	10	c.1180C>T	p.Leu394Phe	reported	/	/	/	/
			10	c.1180C>T	p.Leu394Phe	reported	/	/	/	/
14	early childhood	*EIF2B5*	6	c.806G>A	p.Arg269Gln	reported	maternal	/	/	/
			7	c.915G>A	p.Met305Ile	novel	/	possibly damaging (0.728)	affect protein function(0.00)	disease causing
15	early childhood	*EIF2B3*	8	c.935G>A	p.Arg312Gln	novel	maternal	probably damaging (0.999)	affect protein function(0.00)	disease causing
			9	c.1037T>C	p.IIe346Thr	reported	paternal	/	/	/
16	infantile	*EIF2B4*	6	c.691G>A	p.Gly231Ser	novel	paternal	probably damaging (0.998)	affect protein function(0.00)	disease causing
			12	c.1459C>T	p.Arg487Trp	reported	maternal	/	/	/
17	early childhood	*EIF2B3*	9	c.1037T>C	p.IIe346Thr	reported	paternal	/	/	/
			10	c.1106_1113del	p.S369Sfs*13	novel	maternal	Frameshift		
18	early childhood	*EIF2B5*	7	c.943 C>T	p.Arg315Cys	reported	paternal	/	/	/
			7	c.943 C>T	p.Arg315Cys	reported	maternal	/	/	/
19	infantile	*EIF2B5*	1	c.185 A>T	p.Asp62Val	reported	maternal	/	/	/
			10	c.1518delA	p.E506fs*52	novel	paternal	Frameshift		
20	infantile	*EIF2B5*	9	c.1340C>T	p.Ser447Leu	reported	paternal	/	/	/
			9	c.1340 C>T	p.Ser447Leu	reported	maternal	/	/	/
21	infantile	*EIF2B5*	6	c.806G>A	p.Arg269Gln	reported	paternal	/	/	/
			6	c.806G>A	p.Arg269Gln	reported	maternal	/	/	/
22	juvenile	*EIF2B4*	12	c.1382A>G	P.Tyr461Cys	novel	maternal	possibly damaging (0.559)	affect protein function(0.00)	disease causing
							
			13	c.1565C>T	p.Thr522Met	novel	paternal	probably damaging (1.000)	affect protein function(0.00)	disease causing
23	early childhood	*EIF2B4*	12	c.1306T>A	p.Ser416Thr	novel	paternal	possibly damaging (0.880)	affect protein function(0.00)	disease causing
			12	c.1397G>A	p.Arg446His	novel	/	probably damaging (0.980)	affect protein function(0.00)	disease causing
24	early childhood	*EIF2B4*	10	c.1180C>T	p.Leu394Phe	reported	paternal	/	/	/
			10	c.1180C>T	p.Leu394Phe	reported	maternal	/	/	/
25	early childhood	*EIF2B3*	9	c.1037T>C	p.IIe346Thr	reported	maternal	/	/	/
			/	/	/	/	/	/	/	/
26	infantile	*EIF2B3*	2	c.32G>T	p.Gly11Val	reported	paternal	/	/	/
			2	c.32G>T	P.Gly11Val	reported	maternal	/	/	/
27	juvenile	*EIF2B2*	6	c.818A>G	p.Lys273Arg	reported	/	/	/	/
			8	c.922 G>A	p.val308Met	reported	maternal	/	/	/
28	early childhood	*EIF2B2*	2	c.254T>A	p.Val85Glu	reported	paternal	/	/	/
			8	c.995C>T	p.Ala332Val	novel	maternal	benign (0.450)	affect protein function(0.00)	disease causing
29	early childhood	*EIF2B1*	4	c.328A>G	p.Lys110Glu	novel	/	probably damaging (0.973)	affect protein function(0.00)	disease causing
			4	c.328A>G	p.Lys110Glu	novel	maternal	probably damaging (0.973)	affect protein function(0.00)	disease causing
30	early childhood	*EIF2B2*	2	c.254T>A	p.Val85Glu	reported	maternal	/	/	/
			6	c.818A>C	p.Lys273Gln	reported	/	/	/	/
31	early childhood	*EIF2B4*	4	c.407A>G	p.Gln136Arg	novel	paternal	possibly damaging (0.920)	affect protein function(0.00)	disease causing
							
			4	c.407A>G	p.Gln136Arg	novel	maternal	possibly damaging (0.920)	affect protein function(0.00)	disease causing
32	early childhood	*EIF2B5*	7	c.947G>A	p.Arg316Gln	novel	paternal	probably damaging (1.000)	affect protein function(0.00)	disease causing
			9	c.1352T>C	p.Leu451Ser	novel	maternal	probably damaging (0.988)	affect protein function(0.00)	disease causing
33	early childhood	*EIF2B3*	8	c.965C>G	p.Ala322Gly	novel	paternal	possibly damaging (0.745)	affect protein function(0.00)	disease causing
			9	c.1037T>C	p.IIe346Thr	reported	maternal	/	/	/
34	early childhood	*EIF2B3*	7	c.674G>A	p.Arg225Gln	reported	paternal	/	/	/
			7	c.674G>A	p.Arg225Gln	reported	maternal	/	/	/

#### Constituent ratio of patients and mutations in 5 genes

Among the 34 patients, 12 had mutations in *EIF2B5* (35%), 11 had mutations in *EIF2B3* (32%), 6 had mutations in *EIF2B4* (18%), 4 had mutations in *EIF2B2* (12%) and 1 had mutations in *EIF2B1* (3%). There is a significantly higher incidence of patients with *EIF2B3* mutations compared with Caucasian patients (32% vs. 4%, χ^2^ = 35.136,p<0.001) (Table 2)

**Table 2:** Constitution ratio of patients and *EIF2B1-5* mutations.

Gene	Constitution ratio of patients(%)		Constitution ratio of mutations(%)	
	Reported in Caucasian (n = 250)	Chinese (n = 34)	Reported in Caucasians (n = 150)	Chinese (n = 37)
*EIF2B1*	1	3	4	3
*EIF2B2*	20	12	15	13
*EIF2B3*	4	32	7	19
*EIF2B4*	10	18	17	22
*EIF2B5*	65	35	57	43

Among 37 identified mutations, 16 mutations were in *EIF2B5* (43%), 8 in *EIF2B4* (22%), 7 in *EIF2B3* (19%), 5 in *EIF2B2* (13%), and 1 in *EIF2B1* (3%). (Table 2)

#### Founder mutation in EIF2B3 confirmed

Among the 11 patients who carried mutations in *EIF2B3*, 8 cases (73%, 8/11) harbored at least one copy of the p.Ile346Thr (c.1037T>C). The SNPs of the mutant allele amplified by allele specific PCR were compared among the 8 patients. We found that all of the mutant alleles in these patients shared the same SNPs surrounding the mutation, with t for rs185522, g for rs188710918, t for rs184361839, and c for rs55893665. This suggested the nature of the founder mutation.

#### Natural history

Thirty-four children who were genetically diagnosed were followed up, including 24 males and 10 females. All of the children were unrelated. All of the individuals were children of non-consanguineous parents, except two patients (patient 21 and patient 24). Family history was negative for all but 1 case (patient 4), whose older sister had the similar symptoms and died due to an acute infection of the upper respiratory tract at 2 years of age without definite diagnosis. The phenotype of 34 patients and data of follow-up was described in Table 3.

**Table 3:** The follow-up of 34 gene-confirmed Chinese VWM patients.

**Cases**	**Gender**	**DD**	**Disease onset**		**First visit**		**First Follow-up (2008)**		**Second Follow-up (2010)**		**Third Follow-up (2013)**		**Episodic aggravation**
			**Age**	**Pattern**	**Disease duration (y)**	**Symptoms and signs**	**Disease duration (y)**	**Symptoms and signs**	**Disease duration (y)**	**Symptoms and signs**	**Disease duration (y)**	**Symptoms and signs**	
1	F	_	3y	subacute	1.00 y	II	3.58	IV,S	6.5	V,S	8.83	death	+
2	M	+	1y4m	subacute	0.50 y	III	3	V,S	3.67	death	6.00	death	+
3	M	_	1y6m	acute	1.41	I	3.5	III,S	6.25	IV,S	8.5	V,S	+
4	F	_	2y	acute	5.41	IV	7.33	IV,S	8	death	11.98	death	+
5	M	+	3y8m	acute	0.17	I	2.67	III	4.34	IV	6.67	V	+
6	M	_	6y5m	subacute	0.24	I	1.83	II,S	3.58	III	5.91	V,S	-
7	M	_	4y	acute	0.50	III	1.92	III,S	4.42	III	6.75	IV,S	+
8	M	_	3y9m	acute	0.42	I	1.75	III	5.42	IV	7.75	IV,S	+
9	M	+	1y6m	subacute	0.50	I,S	1.5	IV	4	V	/	/	+
10	F	_	2y5m	acute	0.25	I	0.76	II	/	/	5.50	IV,S	+
11	M	_	4y3m	acute	0.75	I	0.92	II	2.75	III	5.08	IV	+
12	F	_	1y1m	acute	0.09	I	/	/	1.59	death	3.92	death	+
13	M	_	1y8m	acute	0.17	I	UA	UA	/	/	/	/	+
14	F	_	4y6m	acute	7.25	IV	UA	UA	8.75	IV	11.08	V,S	+
15	M	_	2y	acute	1.17	II	UA	UA	1.42	IV	3.75	death	+
16	M	+	4m	subacute	0.09	I	UA	UA	/	/	3.17	death	_
17	M	_	2y3m	acute	0.33	I	UA	UA	0.42	IV	2.75	IV	+
18	M	_	2y6m	acute	0.25	I	UA	UA	UA	UA	1.50	III	+
19	F	+	1y10m	acute	0.17	IV	UA	UA	UA	UA	1.33	death	+
20	M	+	7m	acute	0.17	I,S	UA	UA	UA	UA	0.50	death	+
21	F	_	1y7m	acute	0.17	IV	UA	UA	UA	UA	1.58	death	+
22	M	_	9y7m	subacute	0.17	I	UA	UA	UA	UA	0.33	I	+
23	M	+	5y	acute	0.08	I	UA	UA	UA	UA	2.08	II	_
24	M	_	5y	subacute	0.33	I	UA	UA	UA	UA	2.00	II	_
25	F	+	3y	acute	1.00	III	UA	UA	UA	UA	2.08	V	+
26	F	+	1y5m	acute	0.04	IV	UA	UA	UA	UA	1.00	death	+
27	M	+	7y6m	acute	0.08	II	UA	UA	UA	UA	1.50	II	_
28	M	_	3y	subacute	2.00	I	UA	UA	UA	UA	2.33	II,S	_
29	F	_	2y2m	acute	0.08	IV	UA	UA	UA	UA	1.25	V,S	+
30	F	_	3y	subacute	4.58	II	UA	UA	UA	UA	10.66	IV,S	+
31	F	_	2y6m	subacute	4.33	I	UA	UA	UA	UA	7.75	II,S	_
32	F	_	2y1m	subacute	0.58	III	UA	UA	UA	UA	0.75	III	_
33	F	_	3y7m	aubacute	0.09	I	UA	UA	UA	UA	0.25	I	_
34	F	_	5y	subacute	1.84	II	UA	UA	UA	UA	5.92	IV	+

DD: developmental delay; F: female; M: male; UA: unaviable; /: loss to follow-up; S: seizure; I-V: GMFCSI-V

#### Disease onset

Before the disease onset, developmental milestones were within the normal range in 22 cases (65%, 22/34), and 12 cases had a mild delay in their motor or cognitive development. The average age of disease onset was 2.5 years old (4 months to 9 years 7 months). Twenty-one children met the disease onset age (2–6 years) for the classical early childhood form, 10 children met the onset age (<2 years) for the infantile form, and 3 children met the onset age (6–10 years) for the juvenile form. Twenty-one cases had acute onset (62%, 21/34) after a fever or mild head injury. Thirteen cases suffered subacute onset without obvious incentive. At the first visit, the chief complaint was motor deterioration in all of the children but one, who suffered from convulsions and unconsciousness.

#### Progressive motor deterioration

**Fig 3:**
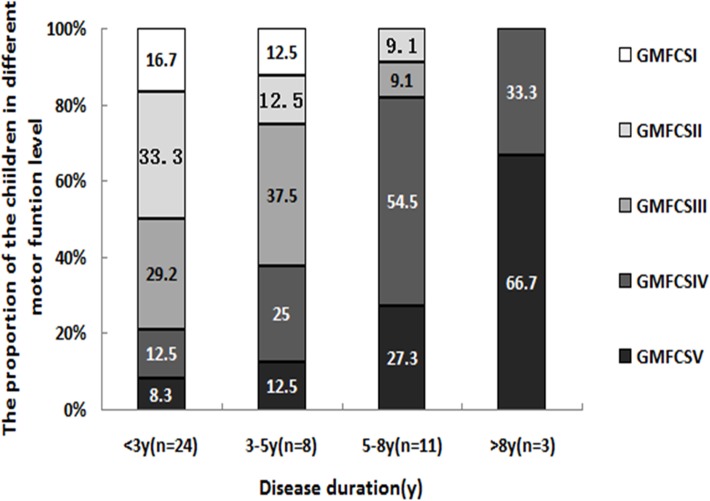
Progression in motor function with prolonged disease duration. Individuals were classified into 4 groups according to their disease duration at the first visit and each follow-up. There were 23 survival cases with disease duration < 3 years, and only 8 patients were surviving at disease duration of 3–5 years. 11 patients survived at disease duration of 5–8 years, and only 3 were still alive >8 years after disease onset. For the surviving children, when the disease duration is less than three years, approximately half of the children still walk independently (GMFCSI-II), 21.7% of the children lost their ability of walking, confined to wheelchair or bedridden (GMFCSIV-V). For the children with disease duration 3–5 years, only 1/4 of the children could still walk independently, and 37.5% of the children were classified as GMFCSIV or V. For the children with disease duration 5–8 years, only 9.1% could still walk independently. All of the survival children with disease duration more than eight years were GMFCSIV or V.

At the first visit, the average disease duration was 1.1 years (5 months to 7 years 3 months). Eleven (91.67%) out of 12 genetically diagnosed cases were being followed up for the first time, 14 out of 17(82.35%) genetically diagnosed cases were being followed up for the second time, and 32 out of 34 (94.12%) genetically diagnosed cases were being followed up for the third time. The average disease duration was 2.8 years (9 months-7 years 4 months), 4.0 years (5 months-8 years 9 months), and 4.4 years (3 months-12 years), respectively. The motor function was progressively impaired in all of the patients. Individuals were classified into 4 groups according to their disease duration at the first visit and each follow-up. There were 28 cases with disease duration less than three years, and 23 of those patients were still alive. Twelve cases had disease duration of 3–5 years, and only 8 of those patients were surviving. Thirteen cases had disease duration of 5–8 years, and 11 patients were surviving. Five cases had disease duration more than 8 years, and 3 of those children were still alive. For the surviving children, when the disease duration is less than three years, approximately half of the children still walk independently (GMFCSI-II), 21.7% of the children lost their ability of walking, confined to wheelchair or bedridden (GMFCSIV-V). For the children with disease duration 3–5 years, only 1/4 of the children could still walk independently, and 37.5% of the children were classified as GMFCSIV or V. For the children with disease duration 5–8 years, only 9.1% could still walk independently. All of the survival children with disease duration more than eight years were confined to wheelchair or bedridden. (Fig. 3)

#### Survival analysis

**Fig 4:**
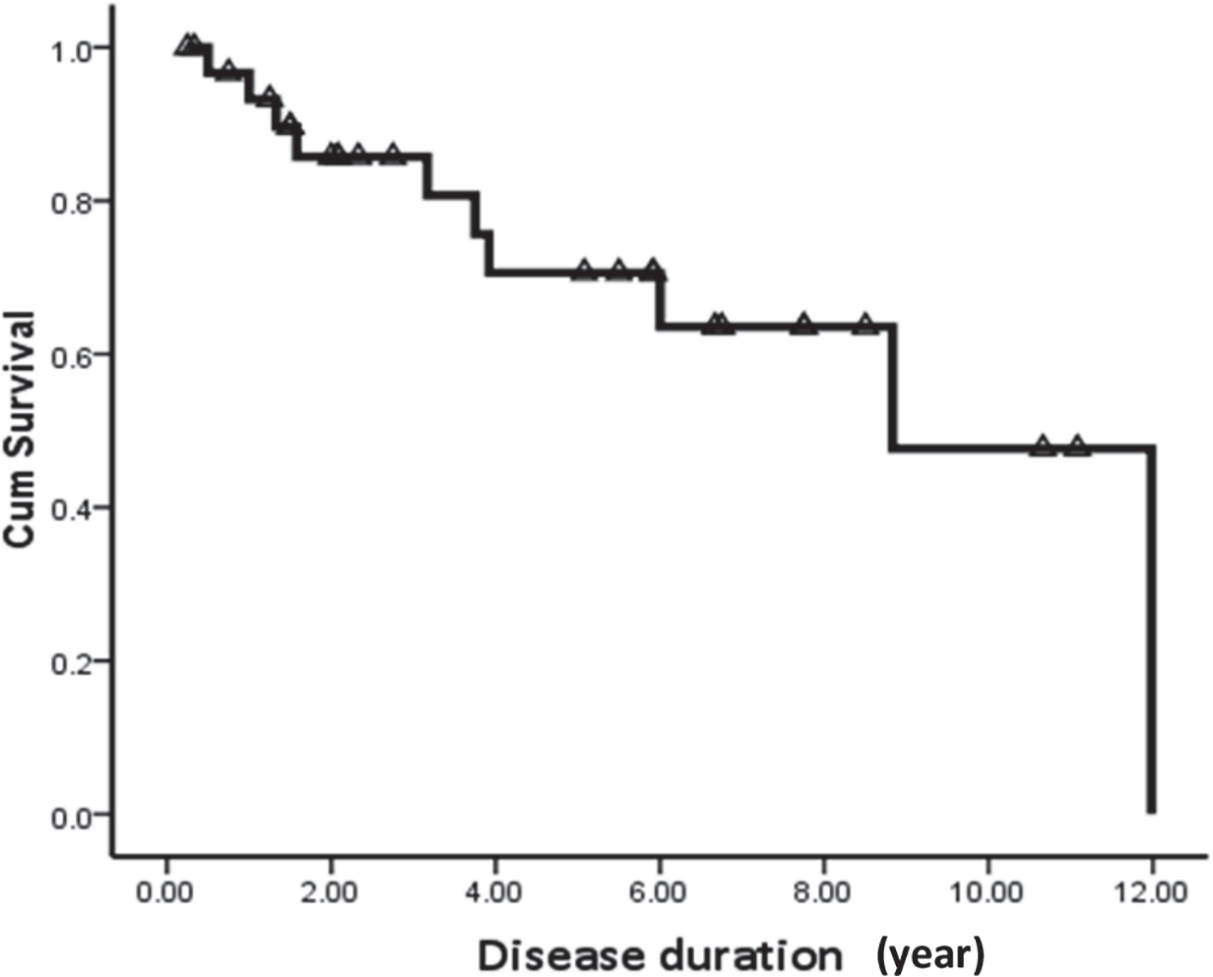
Kaplan-Meier survival analysis. After disease onset, the 1-year survival rate was 93.2%, 3-year survival rate was 80.7% and 5-year survival rate was 70.6%. The 10-year survival rate was 47.7%. The estimated median survival time was 8.83 ± 1.51 years after disease onset.

By the time of the last follow-up, 10 patients (31.2%) died, including 7 infantile cases and 3 early childhood cases. The average age of death in infantile cases was 2.4 years (8months-4years 10months), with average disease duration of 1.2 years (2 months-3 years 8 months). Three early childhood cases died 2.8, 8 and 8.8 years after the disease onset respectively. The age of death was 4.8, 10 and 12 years old respectively. All of the patients died from rapid deterioration after recurrent infections. Survival curve analysis was conducted in the 32 cases at the last follow-up. Ten patients died, and 22 cases were censored. The Kaplan-Meier curve showed that the 1-year survival rate is 93.2%, the 3-year survival rate is 80.7%, the 5-year survival rate is 70.6%, the 8-year survival rate is 63.5%, and the 10-year survival rate is 47.7% (Fig. 4). The median survival time is 8.83 ± 1.51 years.

#### Episodic aggravation

Episodic aggravation precipitated by fever or mild head trauma was observed in 24 cases (70.6%, 24/34), including 9 infantile cases (90.0%, 9/10) and 15 early childhood cases (71.4%, 15/21). The three juvenile cases did not present episodic aggravation during the follow-up. In the condition of acute episode, children showed rapid motor deterioration, which sometimes led to a comatose state. After removing the aggravating cause, there would be gradual improvement but generally could not be restored to the state before the exacerbation.

#### Epileptic seizures

Sixteen patients (50%, 16/32) experienced seizures (with or without fever) during the study, including 12 (75%) early childhood cases and 4 (25%) infantile cases. The seizures usually occurred 1–2 years after the disease onset. The frequency of seizures was once per week to once per year. All of the children showed focal seizures, without status epilepticus. The seizures were usually easily controlled with antiepileptic drugs.

## Discussion

VWM is one of the most prevalent inherited childhood white matter disorders. A wide range of mutations in each of the five subunits of eukaryotic translation initiation factor 2B (*eIF2B1-5*) could lead to this fatal neurodegenerative disease [2]. eIF2B is a complex of five subunits (eIF2Bα, β, γ, δ, and ε). Guanine nucleotide-exchanging factor (GEF) activity to catalyze the eIF2-bound GDP–GTP exchange is necessary for protein translation initiation, and eIF2Bε is the most important subunit [17–19]. The remaining subunits play key roles in the regulation of the GEF activity of eIF2Bε. Gene defects in *EIF2B1-5* inhibit the conversion of eIF2-GDP to eIF2-GTP, resulting in the disruption of protein translation initiation [20–23]. It is suggested that mutant eIF2B resulted in improper activation of unfolded protein response (UPR) in the condition of endoplasmic reticulum stress, which has been proven in fibroblasts or lymphoblasts from VWM patients, and oligodendrocytes from VWM mouse model [24–27]. *EIF2B1-5* are housekeeping genes playing essential roles in various type of cells [28][29], why only the white matter involved is not understood yet.

Since the five causative genes were identified in 2001–2002[3][4], over 250 patients and 150 mutations have been reported. In previous studies from Caucasian patients, the most frequent mutations are in the *EIF2B5* (which contains 57% of all of the mutations), mutations in the *EIF2B4* and *EIF2B2* gene follow, with 16% and 16%, respectively. Mutations in *EIF2B3* account for 7% the mutations, and mutations in *EIF2B1* account for 4% of the mutations [30][31]. In Caucasian patients, approximately 65% of the patients have mutations in *EIF2B5*, 20% have mutations in *EIF2B2*, 10% have mutations in *EIF2B4*, 4% have mutations in *EIF2B3* and 1% have mutations in *EIF2B1* [25] (Table 2).

We confirmed the first case of VWM in China in 2006. To date, a total of 34 Chinese cases have been genetically diagnosed in our pediatric neurological center. The positive rate of gene sequencing in clinically diagnosed VWM children is 94% (34/36). Thirty-seven mutations have been found. Of these mutations, 89% (33/37) are missense mutations, consistent with previous reports [11][32]. Frame-shift and nonsense mutations are rare and often occur in the compound-heterozygous state, which consistent with previous reports [31][31][33–35]. 15 novel mutations were identified, expanding the *EIF2B1-5* mutation spectrum significantly. The most striking finding in our study is that there is a significantly higher incidence of patients with *EIF2B3* mutations compared with Caucasian patients (32% vs. 4%, χ^2^ = 35.136,p<0.001). This finding provided a genetic testing strategy for Chinese VWM patients, which means that *EIF2B5* and *EIF2B3* should be tested first if a traditional sequencing method is used. Since 2013, targeted next-generation exome sequencing has been used in our clinical practice, which helped us to sequence the five genes simultaneously.

In our study, CNVs were not detected in any patients with only one mutant allele or mutation-negative determined by gene sequencing. Shimada S reported a child with phenotype of VWM and multiple malformations who had one copy deletion at the region of 14q24.3 (75,059,408–77,069,618, 2.0 Mb), covering 25 genes including *EIF2B2*, and another heterozygous missense mutation in *EIF2B2* on the other allele [36]. We did not identify any mutations of the *EIF2B1-5* genes in two clinically diagnosed cases (5.6%, 2/36). We hypothesize that mutations located in the non-coding regions of *EIF2B1-5* or other undiscovered causative genes might exist.

We noticed that among the 11 patients who carried mutations in *EIF2B3* (encoding eIF2Bγ), 8 cases (73%, 8/11) harbored at least one copy of the p.Ile346Thr (c.1037T>C) mutation. It was confirmed to be a founder mutation in Chinese patients. Founder mutations are a special class of genetic mutations embedded in stretches of DNA that are identical in all of the people who have the mutation [37]. Everyone with a founder mutation has a common ancestor, the founder, in whom the mutation first appeared. The 8 cases had different surnames. Five of the patients were from northern provinces in China, and 3 were from the southern provinces. Hundreds of years ago, their families probably shared a founder ancestor. p. Thr91Ala in *EIF2B5* has been identified as the founder mutation in Netherlands and North American [38]. The founder mutation might be one of the important causes of the genotypic differences between ethnicities. The mutation p. Arg113His in *EIF2B5* occurs in almost 40% of Caucasian VWM patients [31], and it was thought to be a hot spot mutation. However, it was not identified in the any Chinese patients in our study. Therefore probably it is a founder mutation in Caucasian people.

It is known that episodic aggravation is a prominent clinical manifestation in VWM, but the incidence in infantile and childhood VWM patients is unclear. Labauge P reported that 38% of the adult VWM patients suffered episodic aggravation [39]. In our cases series, 90% of the infantile and 71.4% of the early childhood patients suffered episodic aggravation, suggesting that episodic aggravation is more common in infantile and early childhood VWM. Episodic aggravation was not observed in the three juvenile cases. We cannot rule out the possibility that episodic aggravation appeared later due to the limited number of the cases and the short disease duration.

To assess the motor function, we performed detailed follow-up and analysis. The results suggest that motor function in Chinese VWM children deteriorates rapidly. For the surviving children with the disease duration less than three years, approximately half could still walk independently, and 21.7% of the children score GMFCSIV-V. In the group with disease duration 5–8 years, only 9.1% of the children could still walk independently. In the group with disease duration more than 8 years, all of the survival children were confined to wheelchair or bed. Fogli A estimated motor function in VWM with the Kaplan-Meier method, and the study showed that at 10 years of disease evolution, 95% of infantile cases and 75% of early childhood cases would suffer severe disability (individual required constant supervision, confined to bed or loss of cognitive abilities) [40]. The motor function deteriorated more rapidly in Chinese VWM children, which is probably related to the lack of formal rehabilitation training and social supports after the diagnosis in most of cases.

There were no detailed studies regarding the survival rate and lifespan of VWM patients. It was reported that patients diagnosed in early childhood would die in the second decade of disease evolution [15]. In the present study, 10 patients died by the last follow-up, including 70% (7/10) of infantile cases with an average disease duration of 1.2 years and 14% (3/21) of early childhood cases who died 2.8 years, 8 years and 8.8 years after disease onset, suggesting that earlier disease onset is correlated with shorter survival time. The Kaplan-Meier curve showed that 5-year survival rate was 70.6%, the 8-year survival rate was 63.5%, and the 10-year survival rate was 47.7%. The median survival time was 8.83 ± 1.51 years. The direct causes of death were rapid deterioration in the condition of infections and nutritional disorders, suggesting that nursing conditions, living environment, nutritional support, and social support are the important factors influencing the patients’ survival time.

This study has some limitations. 1) Because the disease currently has no treatment, most of the parents were unwilling to return for visits from a long distance. Telephone contact, which might be biased, is the main way to assess patients’ motor function during the follow-up. And cognitive function evaluation was unavailable. 2) Because of the limited number of cases, we performed an overall assessment of motor function and survival analysis. Grouping the patients according to the age of onset would be better. With the collection of more cases, we will perform more in-depth analyses and more comprehensive follow-up.

## Conclusions

This is the largest sample of children in a VWM follow-up study, which is helpful for a more depth understanding about the children’s natural course of the disease. 15 novel mutations of the *EIF2B1-5* significantly expanded the mutation spectrum of this disease. The proportion of *EIF2B3* mutations in Chinese patients is much higher than the proportion in Caucasian patients, which we assume is related to a founder mutation. There are a small number of clinically diagnosed cases that do not have an *EIF2B1-5* gene mutation, suggesting that there might be other genes involved.
